# To What Extent Memory Could Contribute to Impaired Food Valuation and Choices in Obesity?

**DOI:** 10.3389/fpsyg.2018.02523

**Published:** 2018-12-11

**Authors:** Zhihao Zhang, Géraldine Coppin

**Affiliations:** ^1^Haas School of Business, University of California, Berkeley, Berkeley, CA, United States; ^2^Department of Neurology, University of California, San Francisco, San Francisco, CA, United States; ^3^Swiss Center for Affective Sciences, University of Geneva, Geneva, Switzerland; ^4^Laboratory for the Study of Emotion Elicitation and Expression, Department of Psychology, University of Geneva, Geneva, Switzerland; ^5^Department of Psychology, Distance Learning University Switzerland (Unidistance), Brig, Switzerland

**Keywords:** obesity, memory, value-based decision making, food, hippocampus, episodic memory, semantic memory

## Abstract

Obesity is associated with a diverse array of cognitive and affective deficits, among which impairments in food valuation and choices have received increasing attention. The neural underpinnings of such impairments, however, remain poorly understood, partly because a complete understanding of these processes under normal conditions has yet to be achieved. A rapidly growing literature on the interaction between memory and decision-making has begun to highlight the integral role of memory in decision making especially in the real world, as well as the role of the hippocampus in supporting flexible decision making. Perhaps not coincidentally, altered memory performances in obesity have been well documented, and the underlying neurobiological bases of these memory alterations have also started to be better described, involving pathologies at the biochemical, cellular, and circuit levels. Despite such correspondence, the link between memory impairments and food valuation/choice deficits in obesity has received little attention. In this article, we first summarize the growing empirical support for the relevance of memory for decision making, focusing on flexible value-based decisions. We then describe converging evidence on different forms of memory impairments accompanying obesity. Building on these findings, we formulate a general neuropsychological framework and discuss how dysfunctions in the formation and retrieval of memory may interfere with adaptive decision making for food. Finally, we stress the important practical implications of this framework, arguing that memory deficits are likely a significant contributor to suboptimal food purchase and eating behavior exhibited by obese individuals.

## Introduction

Obesity, characterized by excessive accumulation of body fat as a result of disrupted energy intake and expenditure, shows worldwide epidemic that threatens the health and well-beings of many individuals – life expectancy is decreased by 7 years at the age of 40 (Peeters et al., 2003) and in the United States only, between 280,000 and 325,000 deaths could be attributed to obesity annually (Allison et al., 1999). Rates of obesity worldwide are still rising, and nowadays, there are more overweight than underweight individuals (NCD Risk Factor Collaboration [NCD-RisC], 2016), and the economic cost of obesity for the United States alone is estimated to exceed $275 billion annually (Spieker and Pyzocha, 2016).

Apart from dysfunctions in metabolism, obesity is associated with several cognitive and affective deficits (Elias et al., 2003; Rochette et al., 2016), which have become increasingly well documented. These deficits include those in associative learning (Coppin et al., 2014; Zhang et al., 2014), memory (Gunstad et al., 2006), attention (Cserjesi et al., 2007; Mobbs et al., 2011), and executive functions (Gunstad et al., 2007; Smith et al., 2011). Such deficits have important ramifications as they might exacerbate obesity through their impact on behavior. Out of these diverse behavioral deficits, decision making impairments appear to be robust and consistent across many studies (Davis et al., 2004; Alonso-Alonso and Pascual-Leone, 2007; Jarmolowicz et al., 2014; Amlung et al., 2016), although a large amount of heterogeneity exists under the umbrella of decision-making. Importantly, despite a growing volume of data [see (Wu et al., 2016) for a recent meta-analysis], it remains unclear what the underlying behavioral or neural drivers of such impairments in decision making are.

Among the decision making deficits in obesity in various types of tasks and paradigms, of particular interest are choices regarding food because of their impact on the energy intake. Increasing evidence shows that the consumption of food is driven not only by basic metabolic needs, but also to a large extent by hedonic aspects (Kenny, 2011), highlighting the importance of understanding food-related decision making from a cognitive perspective. In a general sense, decision making can be defined as the cognitive processes that lead to a choice between alternative options or courses of action. A common theoretical framework widely used for modeling and analyzing decision-making assumes that the choice between available options is “on *the basis of a subjective value that [the agent] places on them*” (Rangel et al., 2008), namely a process of *value-based* decision making. The success of such framework lies in its close link to existing theories in economics and psychology (Samuelson, 1948; Tversky and Kahneman, 1981) and its applicability to a remarkably wide range of choice situations in both the laboratory and real life. Furthermore, converging evidence in decision neuroscience also points to the existence of a ‘valuation system’ in the human brain (and probably in brains of other animals as well) including the ventromedial prefrontal cortex (vmPFC) and the ventral striatum (Bartra et al., 2013; Clithero and Rangel, 2014). The activities in these regions apparently encode subjective value signals that seem to be independent with the exact identity or category of the option (Zhang et al., 2017), providing further support for the notion of value-based decision-making.

This framework offers promise in uncovering the mechanisms of suboptimal decision-making with food in obesity. Using neuroimaging techniques such as functional magnetic resonance imaging (fMRI) and positron emission tomography (PET), a well-established literature has shown altered brain reward value responses and dopaminergic activities in obesity (Stice et al., 2008; Volkow et al., 2011; Frank et al., 2012; Babbs et al., 2013; Eisenstein et al., 2013; Guo et al., 2014). These alterations may further be coupled with structural and connectivity changes in the valuation system (Marques-Iturria et al., 2015; Shott et al., 2015). However, rather than an ultimate mechanistic answer to why obese individuals sometimes make suboptimal decisions, these findings should be more appropriately viewed as a step forward, which raises deeper and more challenging questions: If value for certain food items goes awry in obese individuals, why are they not able to get it right in the first place? What goes wrong in the construction and maintenance of value?

In this article, we argue that disruptions in memory mechanisms are likely a key contributor to suboptimal food-related decision making in obesity. To support this claim, the rest of the article is organized as follows. We will first briefly summarize empirical evidence of changes in the valuation system in obesity and point out why this is insufficient for fully understanding both the cause and the scope of changes in decision making in obesity. We will then review the growing literature investigating how memory and hippocampal processes support value-based decision making. This will be followed by a survey of the existing evidence that demonstrates memory impairments in obesity and the accompanying dysfunctions in the hippocampus and related neural circuits. Building on these two lines of research, we will propose a general neuropsychological framework and discuss how dysfunctions in the formation and retrieval of memory may interfere with adaptive decision-making. Finally, we explore the real-world applications of this framework and discuss the possibility that memory deficits contribute to suboptimal food purchase and eating behavior exhibited by obese individuals.

## Suboptimal Food Choices in Obesity: the Need for Going Beyond the Valuation System

A great deal of effort has been devoted to investigating how the reward value of food might be processed differently in obesity. Compared with healthy-weight controls, obese individuals show heightened activation of value-related brain regions in response to food-associated cues (Gautier et al., 2000; Rothemund et al., 2007; Stice et al., 2008; Stoeckel et al., 2008; Dimitropoulos et al., 2012; Rapuano et al., 2016), consistent with the notion that over-valuation of food, especially palatable food, may be a key neurocognitive component of obesity. Meanwhile, a large body of literature reports blunted neural responses during food reward receipt in overweight and obese individuals (Stice et al., 2008; Green et al., 2011; Babbs et al., 2013). In addition, disturbances in value processing is also observed in obesity at the network level, in the form of altered functional connectivity in prefrontal and striatal areas (Stoeckel et al., 2009; Nummenmaa et al., 2012; Garcia-Garcia et al., 2013; Carnell et al., 2014), and such change is not necessarily limited to the processing of food-related stimuli (Verdejo-Roman et al., 2017), potentially reflecting a more general deficit in value-based decision-making. Although most of these studies are cross-sectional and correlational by nature, two recent streams of research provide new evidence of a potential causal link. First, reward responses to food cues in valuation areas such as the striatum, the orbitofrontal cortex (OFC), and the amygdala were found to be predictive of future weight gain by prospective imaging studies (Stice et al., 2010; Demos et al., 2012; Yokum et al., 2014; Sun et al., 2015; Stice and Yokum, 2016). Second, such reward responses were at least partially normalized in patients who underwent bariatric surgery treatment (Shin and Berthoud, 2011; Miras et al., 2012; Faulconbridge et al., 2016).

These advances clearly show the promise of a cognitive approach to obesity, offering a unique perspective complementary to, but distinct from, the traditional approach focusing on metabolism and its related biological factors. It is necessary to consider, however, the gaps that remain to be filled between what we already know and what is needed for the ultimate goal of effective prevention and treatment of obesity. We argue that there are two important avenues for taking this area of research to a new height:

First, it remains poorly understood what the sources of the obesity-related changes in reward responses are. This is by itself a question of great scientific interest. From a biological standpoint, neuroinflammation has recently been proposed to be partially responsible for the cognitive impairments (Miller and Spencer, 2014). In a complementary, cognitive perspective, changes in valuation and its neural correlates are likely the product of changes in a variety of upstream cognitive processes that are coupled to obesity, and the exact identities of these processes remain elusive. For food, knowledge about nutrient qualities as well as past experiences with the food item of interest are likely to be key inputs for value construction. Furthermore, this question also bears significant translational implications for obesity and overeating. Given that options for direct manipulation of the valuation system in the human brain remain extremely limited, behavioral and physiological interventions on the sources of input for valuation may be a more feasible strategy for reliable and consistent changes in food-related choices.

Second, the paradigms used in laboratory studies of decision making with food could be improved to better incorporate the richness of everyday food choices. Typically, many existing studies employ relatively simple, stripped-down choice scenarios, where participants are presented with a fairly small number of options that vary on a limited number of dimensions. While being a useful research strategy, such approach inevitably produces choices that are devoid of many common and important elements in consumer decisions about food in the real world, such as brand knowledge, marketing campaigns, or environmental and contextual influences. Without these factors, how much existing results could generalize to everyday food purchase and eating decisions, which ultimately have the greatest impact on the long-term health and well-being of obese individuals, remain uncertain.

Memory is one attractive point of convergence for these two avenues for future investigations (although of course not the only one). It is likely a critical source of input for the computation and construction of value, and also a key process mediating diverse contextual impacts on decisions in the real world. To lay the foundation for our later discussion on how memory may contribute to impaired food valuation and choices in obesity, we will introduce recent theoretical and empirical advances on the role of memory in decision making in the next section.

## Contributions of Memory to Value-Based Decision Making and Their Neural Bases

Memory and decision making have long been prominent fields of study in psychology and neuroscience (Eichenbaum, 2017; O’Doherty et al., 2017). Despite the significant progress made in both fields, the interactions between memory and decision-making have received little attention until very recently (Weilbacher and Gluth, 2016). In this section, we summarize the emerging evidence showing the crucial roles of memory in supporting value-based decision making. Our discussion will be focused on both new findings and older but potentially overlooked literature that have particular relevance to value-based decisions on food purchase and consumption. We emphasize that memory, which has multiple forms with distinct neural substrates (Squire and Wixted, 2011), could provide important input to different stages of the computations involved in value-based decision making with food.

### Retrieval and Assignment of Value With Memory Mechanisms

In essence, value-based decision making depends critically on the agent being able to construct or retrieve value, the key decision variable, based on the information it has available. A large body of literature on both animals and humans has established that value can be acquired through incremental learning, supported by dopaminergic mechanisms in striatum (Rangel et al., 2008). Note that such process requires repeated experience with the same stimulus and is thus slow and relatively rigid. In contrast, memory mechanisms in the hippocampus provide a complementary implementation of value encoding and retrieval that is faster and more flexible. Such mechanisms may possess better ecological validity, as most decisions outside of a laboratory setting need to be made without the guidance from many past experiences.

In the simplest case, the value of a stimulus is readily available in memory, and can be retrieved to guide decisions directly. In a recent behavioral study (Murty et al., 2016), human participants made decisions about house or face stimuli, each of which representing a certain monetary outcome that they had only a single encounter prior to the decisions. They were able to make optimal decisions based on the value of the stimuli (i.e., choosing the higher value stimulus), but only when they had intact associative memory of the item and the monetary outcome coupled with it. Importantly, such decisions cannot be supported by incremental mechanisms because participants had only experienced the stimulus-reward coupling once and therefore had to rely on the episodic memory of the specific one-shot experiences with the stimuli.

Apart from providing the direct link between value and stimulus identity, memory can also assign value to stimuli with no previously learned value via associative mechanisms. In an influential study by Wimmer and Shohamy (2012), participants first learned to associate pairs of neutral stimuli (S_1_ and S_2_) by their co-occurrence. They then learned the values of one half of these stimuli (S_2_) by experiencing their associations with different amounts of money through classical conditioning. Afterwards, when they were asked to choose between pairs of stimuli drawn from the other half (S_1_) that were never paired with reward and thus had no associated value, they exhibited a robust bias toward those stimuli that were associated with high-value S_2_ stimuli. Again, incremental learning could not be the underlying mechanism for such bias, as participants had not experienced any S_1_-reward association. In support of the involvement of memory mechanisms, hippocampal activation correlates with this decision bias during the S_2_+reward phase, implicating the hippocampus in generalizing the value to the S_1_ stimuli that were otherwise neutral. Decision bias was also related to the functional connectivity between hippocampus and striatum, suggestive of an interaction between memory and valuation. These findings were nicely corroborated by a later animal study reporting the impairment of value generalization through similar higher-order conditioning by hippocampal lesions (Gilboa et al., 2014).

These hippocampal memory mechanisms may not necessarily be mutually exclusive with striatum-based incremental learning; they can influence and interact with each other in a more subtle and nuanced manner. A common strategy to study such interactions is to superimpose a classic incremental learning paradigm with incidental, trial-unique images (Wimmer et al., 2014; Wimmer and Buchel, 2016; Bornstein et al., 2017) or context cues spreading a block of trials (Bornstein and Norman, 2017), so that the influences on value by incremental learning and by episodic memory can be separately modeled and analyzed at both behavioral and neural levels. Collectively, these studies show that episodic memory of past experiences or contexts have a significant impact on the value of available options and, in turn, on choice behavior, which is not captured by incremental learning models (Wimmer et al., 2014; Wimmer and Buchel, 2016; Bornstein and Norman, 2017; Bornstein et al., 2017). Such impact is mediated by neural signatures of memory retrieval, including activities in hippocampus (Wimmer et al., 2014) and in surrounding regions (Bornstein and Norman, 2017), as well as re-activations of value-related neural patterns corresponding to the recalled memory traces (Wimmer and Shohamy, 2012; Wimmer and Buchel, 2016).

### Construction of Value With Memory Mechanisms

So far, we have discussed how memory mechanisms are utilized in retrieving and assigning values to choice options, as well as how they interact with other value learning mechanisms. A common theme in these studies is that value (encoded in and retrieved from memory) is simple and well defined, most often in the form of some fixed amount of monetary reward. In the real world, however, many decisions are more complicated. For instance, one may encounter unfamiliar or novel options in a decision problem, for which there is no pre-experienced or pre-computed value readily available to be retrieved from memory. Under such circumstances, value needs to be constructed on the fly based on certain links that could be drawn between the novel option and past experiences in memory.

A typical way one novel option arises is by combining two or more familiar goods, a key feature of an elegant fMRI study by Barron et al. (2013). Participants were asked to choose between pairs of novel food items, each of which was formed by the combination of two familiar component food types. Critically, such combination had not been tasted together previously, for example tea-jelly and pea-mousse. As a result, participants had to first retrieve the sensory and hedonic experiences of consuming the individual components, imagine the likely experiences of their combinations, and in turn perform online construction of values to guide their choices. Using the technique of repetition suppression (also known as fMRI adaptation), which enables inference of neural representations at the sub-voxel level, Barron et al. (2016) interrogated where and how value was constructed from separate memory traces. Most interestingly, they found indications of simultaneous activations of multiple memories in both the anterior hippocampus and the vmPFC, while the latter region also encoded the chosen value constructed from memories of individual food components during decisions about the novel goods. These results extended previous findings on value encoding in the vmPFC, showing that value signals in this region could also result from the integration of memories retrieved in the hippocampus. Furthermore, this study bears particular practical relevance to everyday food choices in a modern world, where numerous food manufacturers, retailers, and restaurants constantly bombard consumers with opportunities of novel eating experiences that stem from more familiar components.

One important question that remains open is the nature of those memories being integrated to construct value in light of the framework of multiple memory systems (Squire, 2004; Squire and Wixted, 2011). On one hand, one could draw information from episodic/autobiographical memory and therefore focus on the more experiential and contextual aspects, for example “When was the last time I had a mousse and how did I feel about its taste and texture?” In this case, associative mechanisms similar to those summarized in the previous section are likely to be employed, with hippocampus and vmPFC serving a central role in retrieval and integration (Benoit et al., 2014; Madore et al., 2016; Gershman and Daw, 2017). On the other hand, one may rely on the abstract conceptual knowledge about the options and its components, which falls under the system of semantic memory, for example “How much sugar and fat does this ice cream cone contain and is it good for my health?” This kind of semantic knowledge is a powerful tool for efficient generalization by utilizing commonalities between novel and known stimuli (Shea et al., 2008; Kumaran et al., 2009), without the need for first-hand experiences of everything. For many real world decisions with numerous dimensions, it is a very attractive strategy for making such complex decisions tractable by reducing them to the evaluation and integration of a few core attributes that one possesses relevant knowledge about. For food-related decisions, both caloric density (Tang et al., 2014; Suzuki et al., 2017) and macro-nutriments (Suzuki et al., 2017; DiFeliceantonio et al., 2018) are key determinants of value. It has recently been shown that conceptual beliefs about elemental nutritive attributes of food, including carbohydrates, protein, fat, and vitamin content, are represented and integrated in the orbitofrontal cortex (OFC), an area adjacent to the vmPFC, to compute the overall value of a food item (Suzuki et al., 2017). But caloric density is also associated with responses in the vmPFC, even though participants cannot correctly estimate the food items’ caloric content (Tang et al., 2014; Suzuki et al., 2017). Interestingly, this effect may vary with different macro-nutriments: DiFeliceantonio et al. (2018) recently showed that participants are better at estimating caloric density of fat foods compared to carbohydrate or fat + carbohydrate foods, with such effects associated with the functional connectivity between the vmPFC and the fusiform gyrus.

More broadly, value computation for many realistic decisions, whether they are about familiar or novel items, may need the input from both episodic and semantic memory. A general theoretical model that could be applied to such cognitive processes mandates that the sequential sampling of evidence from memory serves as the basis of value construction (Shadlen and Shohamy, 2016). Viewed from this perspective, value is not instantaneously constructed from all relevant pieces of information. Instead, value is updated by the incorporation of every new piece of evidence generated from a sequential process of memory sampling/retrieval. Analogous to the drift diffusion model widely used in studies on perceptual decision-making (Gold and Shadlen, 2007), once this sequentially updated value reaches a certain threshold, a decision will be committed. One recent fMRI study provided initial empirical support to this model by showing that hippocampal activation was related to the time it took the participants to make a choice between two familiar food items, presumably reflecting the amount of information sampled from memory (Bakkour et al., 2018). Similarly, hippocampal lesion patients also showed deficits making binary choices between familiar food items, but had performance comparable to healthy controls on a number-comparison control task, again highlighting the role of hippocampus in value construction from memory (Enkavi et al., 2017).

### Generating the Choice Set With Memory Mechanisms

Previously, we have discussed how memory can contribute to value-based decision making when the available options are clearly specified. This constraint is readily fulfilled by most laboratory studies on decision making, but often does not hold in choices made in the real world. Imagine that, after being seated at a table in a restaurant, you are asked by the waitress, “Can I get you something to drink?” In this case, what you can choose from (i.e., the “menu” for this choice) is not explicitly communicated. Despite such apparent ambiguity, most people have no difficulty making a choice, without having to refer to the actual beverage menu or asking the waitress what are available.

More formally, this decision can be modeled as a two-stage process: constructing the choice set from memory, followed by a decision over the items in the set (Shocker et al., 1991). Examples alike are abundant in daily life, ranging from picking a vacation destination to deciding on what grocery products to buy before arriving at the store. A critical feature of such decisions is that memory places a constraint on choices: an option can only be chosen if it is successfully recalled. In this case, the effect of memory on choice is different from the assignment and construction of values. Indeed, a series of behavioral studies demonstrated that changes in the ease of retrieval of certain options have a significant effect on such open-ended choices, while the preferences remain unchanged (Nedungadi, 1990; Posavac et al., 1997; Shapiro et al., 1997; Posavac et al., 2001).

This type of decisions is largely neglected by studies of cognitive neuroscience so far, but has been a popular research topic in consumer behavior (Alba et al., 1991), because of its close link to advertising and marketing (Krishnan, 1996). However, the source of the memory necessary to generate the choice set can be much more diverse than marketing campaigns, such as word of mouth, general knowledge learned from formal schooling, facts and ideas acquired from news media, and even cultural and religious beliefs. One common type of decisions with self-generated choice sets involve choosing an alternative from a given category (e.g., different kinds of fruit, brands of smartphones, etc.), for which semantic memory provides the most suitable input for the choice set. In addition to semantic memory, episodic memory of past experiences (e.g., “What kinds of fruit were available in the farmers market last week?”) or choices (e.g., “Which restaurant did we go to when John visited?”) may also contribute to the generation of choice sets, albeit likely in a more context-dependent manner. Yet, the arbitration of what information to retrieve from semantic and episodic memory, as well as the neural mechanism supporting the integration of memory and value, still need to be clarified.

Lastly, it is worth noting that memory-based constraints on choice sets may still apply even when all relevant information is physically present (e.g., standing in front of a shelf full of different breakfast cereal brands), as most decision makers may not have the motivation or the cognitive resources to process it (Park et al., 1989), leading their attention and/or search effort to be largely guided by memory (Hutchinson and Turk-Browne, 2012). In a related setting, Gluth et al. (2015) showed that options for which the spatial locations were remembered were more likely to be chosen. In parallel, this bias was linked to the effective connectivity between hippocampus and vmPFC, a key valuation area in the brain. Hence, the constraint on the choice set by memory is likely to have a far-reaching effect on many real world decisions than just the ones that require an internal choice set to be generated completely from memory.

## Memory Impairments in Obesity

We now move on to discuss empirical evidence showing memory impairments in obesity, as well as their neural signatures. Although the study of memory has a remarkably long history, the investigation on memory impairments in obesity is still a burgeoning field involving exciting research efforts with both human and animal studies. Notably, while the two streams of research serve the common goal of unraveling the complex interactions between eating behavior, energy dysregulation, and memory functions, they have largely employed different methodologies due to practical constraints. Unlike animal studies where manipulations of energy intake, diet, and even neural activities are possible, human studies on this topic often involve cross-sectional comparisons between obese individuals and healthy controls. As a result, causal inference is usually not possible. Despite such limitations, a common thread has emerged from accumulating data, indicative of an association between obesity, suboptimal memory performances, and functional disruptions in neural circuits supporting important aspects of memory in humans.

### Episodic Memory Impairments in Obesity

Episodic memory is defined as the memory for specific events or episodes in one’s past experience (Tulving, 1972). Following traditions dating back to Ebbinghaus (2013), a widely used way to probe episodic memory is through learning and recalling items on a pre-defined word list, which have also been employed by a majority of publications on obesity and episodic memory. In one of the earliest studies, Cournot and colleagues reported higher body mass index (BMI) was significantly associated with lower score at the delayed memory recall test, after adjusting for demographic variables (age, sex, education level, and region of residence) and physical activity (Cournot et al., 2006). Similarly, Gunstad and colleagues also found an association between poorer performance in the verbal list-learning task and higher BMI (Gunstad et al., 2006). Using a sample with a wide age range (21–82 years), they also tested the possible interaction between BMI and age on memory performance and found no evidence of such interaction. This finding shows that lower memory performance in obese individuals are not likely an artifact of higher age, and that age-related and obesity-related memory decline may have separable etiologies. Extending the results from these two studies, Dore et al. (2008) replicated the inverse association between memory and body weight status (measured by waist circumference and waist/hip ratio, WHR, rather than BMI, in their study), and also showed that such association was attenuated with adjustment of physical activity levels. Importantly, these studies all used relatively large samples, ranging from several hundreds (Gunstad et al., 2006; Dore et al., 2008) to more than two thousand (Cournot et al., 2006), which lends strong statistical support for their findings.

Building on these groundbreaking findings, a recent series of research sought to examine if such negative associations could be reversed following interventions that reduce body-weight, such as diet and bariatric surgery [for a recent review and meta-analysis, see (Veronese et al., 2017)]. Across multiple studies, beneficial effects of bariatric surgery on memory performance on the same verbal list-learning task were found, accompanying post-operative weight losses (Gunstad et al., 2011; Miller et al., 2013; Alosco et al., 2014a; Spitznagel et al., 2014). These studies also replicated memory impairments in obese individuals before the surgery. Along the same line, another group of studies also demonstrated a moderately positive effect of dietary interventions on memory in obese/overweight individuals (Siervo et al., 2011; Boraxbekk et al., 2015). Overall, such prospective or experimental studies took an important step forward from cross-sectional studies comparing memory performances between groups of participants with different body weight status, and substantially strengthened the link between episodic memory functions and obesity.

As a probe to episodic memory, the verbal list-learning task is a paradigm easy to implement and analyze. However, such simplicity also makes it challenging to connect this paradigm to the much more complicated contents of episodic memory in real life that may be related to food choice and consumption. As a result, there have been attempts to take advantage of novel paradigms for episodic memory that capture richer dimensionalities to test and validate memory deficits in obesity. In a pair of behavioral and neuroimaging studies (Cheke et al., 2016, 2017), Cheke and colleagues employed a so-called “Treasure-Hunt task” that directly tests the three key elements of episodic memory – object recognition, location, and temporal order (“what-where-when”) – as originally defined by Tulving (1972). In a group of young adults, performance in all individual elements of this task was negatively associated in BMI (but see Cole and Pauly-Takacs, 2017). In the follow-up fMRI study, when compared with healthy controls, obese participants showed reduced neural activities in medial temporal regions including hippocampus and parahippocampal gyrus, as well as the angular gyrus and parts of the prefrontal cortex, a network implicated in memory retrieval. Being the first studies investigating memory deficits in obesity that broke away from the tradition of verbal list-learning tasks, these results were still exploratory and should be interpreted with caveat (e.g., the behavioral differences were not fully replicated in the neuroimaging study despite the significant between-group differences in neural activities). Still, the work by Cheke and colleagues highlighted the utility of aiming for better ecological validity while still maintaining experimenter control over what participants learn and retrieve.

In a similar vein, autobiographical memory tasks that ask participants to retrieve information about past experiences in their daily lives also enable researchers to test episodic memory abilities (Cabeza and St Jacques, 2007). This subtype of episodic memory may also exert a considerable impact on eating behavior (see Section 2.2). This may also be true for false memories, i.e., memories of events that never occurred (Bernstein and Loftus, 2009; Chen et al., 2017; Howe et al., 2017). However, to our knowledge there has been no behavioral or neuroimaging study that examines autobiographical memory performances in obesity. Furthermore, recent evidence implies that list-learning tasks and autobiographical memory tasks recruit different neural substrates, challenging the degree to which episodic memory is a unified construct (Chen et al., 2017). Therefore, future studies are needed to address potential impairments of autobiographical memory in obesity and how well they correspond to known deficits in verbal list-learning tasks.

More generally, for most if not all of the episodic memory tests in existing studies, the content of information that participant needed to encode into and retrieve from episodic memory had little relationship with food and eating behavior. Because of the centrality of energy intake and eating behavior in obesity and related disorders, the question of domain specificity of any associated cognitive deficits is an important one and bears crucial implications for how such deficits affect behavioral output. This gap in literature should be filled by future studies.

### Semantic Memory Impairments in Obesity

Contrary to episodic memory, semantic memory is defined as memory for facts, concepts, beliefs not attached to specific events or episodes, or more broadly as one’s world knowledge (Tulving, 1972). Semantic memory is believed to be one of the cognitive processes that are uniquely human, and it guides a large variety of our everyday activities (Binder and Desai, 2011). As previously mentioned, clinical assessments of semantic memory capabilities usually involve tasks like semantic fluency and categorization. Generally, semantic memory in obesity have so far received less attention than episodic memory, and only a small number of studies included semantic memory assessments as part of a bigger battery of cognitive tests. With limited data, evidence regarding the existence of semantic memory impairments in obesity is inconclusive. Several studies reported significant differences between obese and control participants, pointing to a decline in semantic memory retrieval accompanying obesity. For instance, a mostly obese sample of patients with metabolic syndrome showed lower scores in an animal-naming semantic fluency task (Segura et al., 2009). Similarly, a large cohort study reported significant lower verbal fluency (a composite measure of semantic and phonemic fluency) in obese and overweight participants, and in metabolically abnormal participants (Singh-Manoux et al., 2012), although information about semantic fluency itself was not made available. On the other hand, a couple other studies reported no significant difference in semantic fluency (using animal naming tasks as well) between obese and controls (Boeka and Lokken, 2008; Alosco et al., 2014a).

We argue that studies on semantic memory in obese individuals are still inadequate, and that extant results should not be interpreted as weak or even no association between obesity and semantic memory. First, similar to episodic memory tests conducted on obesity-related studies so far, the category chosen for semantic fluency tasks is usually limited to animal, which may have limited generalizability and also has little to do with food and eating behavior. It is of great scientific interest to specifically test the semantic memory about food-related items and categories in obese and overweight individuals, as well as comparing it with knowledge with other common categories and domains. Second, while analysis of performances in the semantic fluency tasks has almost always focused exclusively on the number of correct items generated within the time limit, there is much richer information that remains unexplored. Semantic memory is thought to be organized in complex tree- or network-like structures (Jones et al., 2015), and retrieval processes in the semantic fluency task can be modeled as a random walk on such underlying structures (Abbott et al., 2015). Mere comparison of the number of items generated may fail to uncover important changes in semantic memory structure associated with obesity. Third, most extant studies had relatively small sample sizes and were unable to sufficiently control for heterogeneities in comorbidity conditions, adding to the difficulty of identifying potentially subtle but impactful differences.

### Neural Correlates of Memory Impairments in Obesity

As summarized above, although both the extent and the nature of memory dysfunctions in obesity have yet to be fully determined, suboptimal memory performances in obese individuals have been reported in a variety of tasks. In parallel with these behavioral findings, a growing literature has also documented alterations or abnormalities in the anatomy and/or physiology of brain regions known to underlie memory functions, lending further support and mechanistic understandings to deficits shown in behavior.

The earliest evidence for such alterations came from three large-scale neuroanatomical studies. Obesity, as measure by BMI or WHR, was reported to be associated with smaller global brain volume (Ward et al., 2005), temporal lobe atrophy (Gustafson et al., 2004) or decrease in hippocampal volumes (Jagust et al., 2005), with the latter two focusing on aging samples. The same association was later confirmed on young adults as well (Mueller et al., 2012). Other studies further explored other age or specific gender groups (Walther et al., 2010), the use of different measures of adiposity and metrics of anatomy (e.g., voxel-based morphometry, or VBM), as well as potential anatomical changes in brain regions outside of the temporal lobe [for a review, see (Willette and Kapogiannis, 2015)]. Of interest to our discussion here are reports showing that higher BMI also predicted less gray matter volume in a limbic lobe ROI that included hippocampus and surrounding areas in children and adolescents (Alosco et al., 2014b), further confirming that such association was not specific to an aging population. A recent longitudinal study also showed that change in BMI over a 5-year period was specifically associated with change in hippocampal volume but not with any other ROIs (Bobb et al., 2014), highlighting the centrality of structural changes in hippocampus in obesity. In addition, several studies pointed to a negative association between BMI or fat mass and less gray matter volume in parts of the prefrontal cortex, (Pannacciulli et al., 2006; Ho et al., 2010; Kurth et al., 2013), especially its medial wall, known to be important in memory retrieval and integration.

Findings using other imaging modalities have further expanded these results. With diffusion-weight imaging that provides information about local movement of water molecules, Alkan et al. (2008) showed altered fluid distribution in a range of brain regions in obese individuals, including hippocampus and middle temporal cortex. Volkow et al. (2009) demonstrated an inverse association between BMI and prefrontal metabolic activity using positron emission tomography (PET). Geha et al. (2017) found that functional connectivity between different brain regions was reorganized in obesity, involving parts of prefrontal cortex and anterior hippocampus.

In summary, there is converging evidence supporting the notion that obesity is associated with changes in a core brain network that is crucial for memory functions. It should be noted that memory impairments and brain changes in obesity have largely been studied separately so far. The issue is alleviated to some degree by the fact that the neural circuits mediating episodic memory and semantic memory have been reasonably well delineated (Binder and Desai, 2011; Moscovitch et al., 2016; Ralph et al., 2017). Still, the link between these two levels of analysis needs to be more firmly established in future studies that directly test their correspondence.

## An Integrative Framework for Contributions of Memory Mechanisms in Suboptimal Food Choice in Obesity

All cognitive processes serve the ultimate goal of more adaptive behavior, and memory is no exception. Identification of memory impairments in obesity is an important research topic, but its actual impact on obese individuals can only be understood if we know how such impairments translate to behavior. In this section, we seek to provide a synthesis that combines (1) how information retrieved from memory can act as the essential input for different stages of value-based decision making and (2) the empirical evidence for memory impairments and accompanying neural changes in obesity. To achieve this goal, we propose a unified neuropsychological framework capable of explaining and predicting how memory impairments in obesity map onto altered decision making, in particular the ones involving food valuation and choice. Such framework will be beneficial for organizing existing findings under a common theme of understanding maladaptive choice behavior, as well as for identifying gaps and holes that future studies can be planned to fill. More importantly, this framework will also be valuable for guiding the discovery of more effective interventions and public policies addressing overeating and associated decision making vulnerabilities.

### Effects of Memory on Eating Behavior

Connecting memory and eating behavior is an important step toward better understanding of how cognitive deficits in obesity lead to problematic behavior. A series of pioneering studies from Higgs and colleagues have beautifully illustrated the utility of such approach. In this line of research, Higgs (2002) focused on how memory for recent eating experiences influences subsequent intake of food. Prompting female participants to recall what they had eaten for lunch today caused them to eat less in an experimental session, compared to participants who received no cues or a cue for the lunch yesterday (Higgs, 2002). Distraction during lunch by television watching increased the intake of afternoon snacks in female participants, which was caused by interfering with formation of memory for the eating experience during lunch (Higgs and Woodward, 2009). Similarly, a different manipulation on attentiveness during lunch (i.e., focusing on food vs. a newspaper article about food) lowered snack intake afterwards, while the amount of snack intake was negatively associated with rated vividness of lunch memory (Higgs and Donohoe, 2011). The effect of (episodic) memory for a meal and interference of memory encoding by a distractor task on subsequent food intake was further replicated in other contexts and with different manipulations (Oldham-Cooper et al., 2010; Mittal et al., 2011) [for a review and meta-analysis, see (Robinson et al., 2013)]. Furthermore, these behavioral studies were nicely complemented by the findings that amnesiac patients with hippocampal damage were not able to form and retrieve memory for recent eating and, as a result, showed excessive food intake when offered multiple meals (Rozin et al., 1998), despite intact sensory specific satiety (Higgs et al., 2008).

So far, this fruitful line of studies has largely focused on episodic memory about a particular event of eating and how it affects the amount of food intake shortly after. In fact, as we discussed in section “Contributions of Memory to Value-based Decision Making and Their Neural Bases” of this review, there are many more channels through which memory (not necessarily limited to memory about an earlier meal) could play an important role in choices regarding food intake. In addition, despite the abundance of behavioral data from healthy participants and lesion patients, it remains unclear through what computational and neural mechanisms that memory changes subsequent food intake. Incorporating these findings and extending them through the framework of value-based decision making will take advantage of the well-understood neural circuitries supporting valuation and choice.

### Memory Impairments and Suboptimal Food Choices in Obesity

**FIGURE 1:**
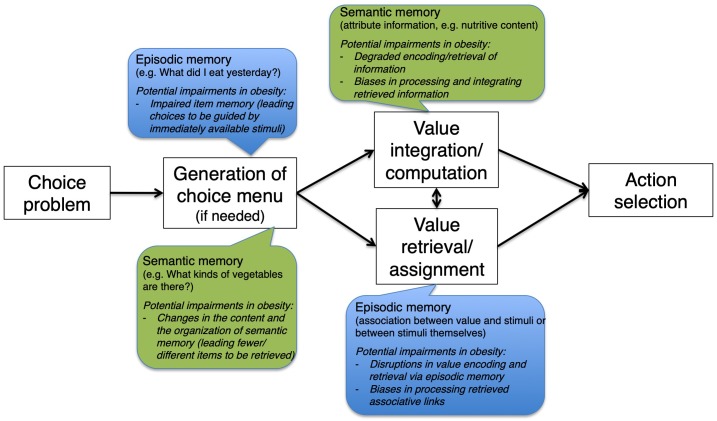
A schematic presentation of the proposed general neuropsychological framework for how memory impairments could lead to suboptimal food-related decision in obesity.

To establish a general neuropsychological framework for suboptimal food choices in obesity that is driven by memory impairments, we propose the following taxonomy based on our previous discussion and raise a series of research questions that are worth pursuing in future studies (Figure 1). First, in the simplest situation where value can be directly retrieved from episodic memory based on one-shot experience (see section “Retrieval and Assignment of Value With Memory Mechanisms”), suboptimal valuation of certain food items could result from either a bias or an increased level of noise around the true value. Qualitatively, this is similar to impairment in recalling specific items from a previous learned word list. It would be helpful to test the encoding and retrieval of value in episodic memory (Murty et al., 2016) by obese participants and compare their performance with that of healthy controls as well as the generalizability of such impairment (i.e., whether it is restricted to the value of particular types of food). Alternatively, it is also possible that, while the value can be faithfully retrieved from episodic memory, the valuation area is unable to process it correctly. A neuroimaging experiment based on existing insights would be able to tease these apart: given that increased coupling was shown between hippocampus and valuation areas during such choices (Wimmer and Shohamy, 2012), it would be of great interest to test if such connectivity is weakened in obese individuals, causing the deficits in valuation and subsequent choices.

Second, when value of a food item needs to be constructed from memory, a number of different scenarios could give rise to valuation deviating from what it should be. One possibility is that the integration of multiple memory traces mediated by hippocampus (Barron et al., 2013) becomes problematic, such that certain traces receive disproportionately higher weights than others. If the choice needs to be guided more by semantic memory about a number of attributes of the item in consideration, dysfunctional communications between valuation areas like the vmPFC, OFC, and striatum and semantic memory areas including the inferior frontal gyrus and the temporal lobe could be a key driver of suboptimal choices. For food-related decisions in obese individuals, it is of particular interest to investigate if abnormalities exist in the representation of major nutrient contents of food (Suzuki et al., 2017). Again, existing experimental paradigms that have proved successful in uncovering the cognitive and neurocomputational mechanisms in healthy individuals would be a useful reference and baseline for investigations on obese participants and the like.

Third, for food-related decisions where the choice set needs to be assembled on one’s own (e.g., picking a nearby restaurant to go to from memory) or be pruned for efficiency (e.g., choosing from an overload of breakfast cereal brands), semantic memory of specific categories and exemplars is a vulnerable point where suboptimal choices can originate from. In particular, if the organization of such memory (including the identity of the concept nodes, the structure of the network, and the strength of links between nodes) is altered (Abbott et al., 2015), it is conceivable that the composition of the internal choice could deviate from what a healthy individual would construct. An example would be someone who always thinks of soda and energy drink instead of more healthy beverages like water or tea when thirsty. In addition, there could also be differences in the sensitivity to information from the external environment, such as marketing campaigns and advertisements, between obese and healthy individuals, such that certain information is preferentially absorbed into semantic memory, which further influences memory-driven choices later.

Such framework helps us better able to leverage the wealth of knowledge garnered from multiple decades of basic science research on memory and decision making. We believe that this integrative framework will not only prove beneficial for relevant research, it also has the potential to shed new light on evaluating and improving behavioral interventions addressing obesity. In particular, it calls for the identification of causes of a known behavioral problem (suboptimal food valuation and choices) at a more up-stream level by examining one of its key inputs (memory). Rather than targeting valuation and choices itself, creating an informational environment that protects vulnerable individuals from pitfalls created by memory could be a more effective strategy.

## Conclusion and Final Remarks

In this article, based on recent advances in decision neuroscience and in the study of memory impairments in obesity, we propose an integrative framework on how much memory impairments could contribute to suboptimal food valuation and choices in obesity. We argue that, by placing obese individuals back into the rich, dynamic environment constantly shaping and changing their memory, we can obtain a better insight on their eating behavior by considering how memory affects their everyday decisions with food. We hope that the proposed framework can spark new interest in the intersection between memory, valuation, and obesity.

Finally, it should be noted that we by no means claim that the proposed framework explains all, or even a majority of, decisions with food. For example, we do not discuss the heavily debated topic of food addiction (Gearhardt et al., 2011; Hebebrand et al., 2014; Volkow et al., 2017), nor do we attempt to address the effect of lifestyle, genetics, personality traits (e.g., impulsivity and self control capabilities), and environmental factors (e.g., stress) on eating (Nederkoorn et al., 2006; Hare et al., 2009). Moreover, we do not mean to imply that attentional bias does not play an important role. On the contrary, we believe it is likely that attentional bias may exert further impact on memory processing above and beyond impairments in memory itself, which could exacerbate suboptimal food decisions [for this topic, see (Higgs and Spetter, 2018)]. Rather, we argue that our framework can potentially explain a non-trivial proportion of suboptimal food-related decision making in obesity that other models may have difficulty accounting for. Given the pervasiveness of the obesity epidemic and the lack of effective interventions, even a small step toward a more complete picture of the mechanistic underpinnings of the impairment can be beneficial.

## Author Contributions

ZZ and GC conceived the project. ZZ wrote the manuscript with GC’s input.

## Conflict of Interest Statement

The authors declare that the research was conducted in the absence of any commercial or financial relationships that could be construed as a potential conflict of interest.
